# A comparative study of portoenterostomy and choledochojejunostomy in New Zealand rabbits under immunosuppression

**DOI:** 10.3389/fsurg.2025.1660016

**Published:** 2025-12-18

**Authors:** Jiuzheng Sun, Haiming Zhang, Hao Huang, Wei Qu, Zhi-Jun Zhu

**Affiliations:** 1 Liver Transplantation Center, National Clinical Research Center for Digestive Diseases, Beijing Friendship Hospital, Capital Medical University, Beijing, China; 2 Department of Hepatobiliary and Pancreatic Surgery, Central Hospital Affiliated to Shandong First Medical University, Jinan, China; 3 Laboratory for Clinical Medicine, Capital Medical University, Beijing, China; 4 State Key Laboratory of Digestive Health, Beijing, China; 5 Beijing Key Laboratory of Tolerance Induction and Organ Protection in Transplantation, Beijing, China

**Keywords:** portoenterostomy, choledochojejunostomy, living donor liver transplantation, radical surgery for hilar cholangiocarcinoma, surgical technique

## Abstract

**Background:**

This study aims to evaluate the safety and feasibility of performing portoenterostomy in rabbits under immunosuppressive therapy.

**Methods:**

49 New Zealand rabbits were randomly divided into two groups: the experimental group (*n* = 24) underwent portoenterostomy, while the control group (*n* = 25) received traditional choledochojejunostomy. Postoperatively, all rabbits were administered oral methylprednisolone and cyclosporine A. Four rabbits from each group were sacrificed on postoperative days 30, 60, and 90. Primary outcomes included postoperative mortality, morbidity, and pathological changes at the anastomotic site. Secondary outcomes encompassed biochemical indicators such as ALT, AST, ALB, ALP, GGT, DBIL, TBIL, TBA, BUN, and Scr.

**Results:**

In a study comparing portoenterostomy and choledochojejunostomy, 49 rabbits underwent surgery (24 in the portoenterostomy group and 25 in the choledochojejunostomy group). The postoperative mortality rates were 4/24 in the portoenterostomy group and 6/25 in the choledochojejunostomy group. Changes in serum bilirubin and creatinine levels suggested the occurrence of anastomotic stricture, cholangitis, and renal dysfunction in some rabbits. The portoenterostomy group showed less severe anastomotic stricture compared to the choledochojejunostomy group, and the use of immunosuppressive agents did not negatively affect the healing process. Pathological observations indicated good healing outcomes within 1 to 3 months postoperatively, despite the presence of inflammation and scar formation.

**Conclusions:**

The study demonstrates that small portoenterostomy performed under immunosuppressive therapy is a safe, simple, and technically feasible surgical approach.

## 1 Introduction

With continuous advancements in liver transplantation techniques, the shortage of donor livers has become a critical bottleneck restricting its development (1–8). Particularly in Asia, where awareness of organ donation remains relatively low, many patients with end-stage liver disease either pass away while waiting for a suitable donor liver or miss the opportunity for transplantation due to disease progression. This urgent situation has directly driven the development of living donor liver transplantation (LDLT) (9–11). However, anatomical variations in the biliary tree of some donors can result in multiple small bile ducts on the donor liver cut surface, necessitating intricate anastomotic procedures.

In pediatric LDLT, where only the left lateral lobe or an even smaller portion of the adult donor liver is used, the donor liver cut surface often contains two or more small bile duct stumps, which are typically of very small diameters. In recent years, laparoscopic donor hepatectomy has made significant advancements, but the difficulty in performing intraoperative cholangiography during laparoscopic procedures sometimes results in high-level bile duct transections, leading to multiple small bile ducts on the cut surface. For donor livers with multiple small bile ducts, reconstruction can involve either portoenterostomy or choledochojejunostomy (12–18). When the small bile ducts are spaced far apart, the complexity of bile duct reconstruction increases significantly, requiring individual bile duct-jejunal anastomoses. However, bile ducts that are too small can lead to complications such as bile leakage, stricture, and cholangitis after anastomosis, which not only hinder patient recovery but may also necessitate re-transplantation.

The Kasai procedure, developed by Japanese scholar Morio Kasai in 1968, was initially designed for treating uncorrectable biliary atresia. Today, it is primarily used to manage congenital biliary atresia in children to prolong their waiting time for liver transplantation (19–26). Similarly, portoenterostomy has been applied in the surgical management of hilar cholangiocarcinoma (27–32). After the resection of hilar cholangiocarcinoma, multiple bile ducts and short residual bile duct stumps are often present. Traditional choledochojejunostomy techniques cannot provide sufficient length for the bile duct stumps, and complications such as bile leakage, stricture, and high mortality rates are common. In contrast, portoenterostomy offers complete tumor resection while reducing the incidence of these complications and mortality rates.

In LDLT, due to the limited range of donor selection, some donors may present with biliary variations. This is especially true in pediatric liver transplantation, where parents serving as donors may provide only the left lateral lobe or a specific liver segment. The liver cut surface often contains multiple small bile ducts with small diameters. In some cases, the spacing between bile ducts is too wide to allow for repair or reconstruction, necessitating individual bile duct-jejunal anastomoses. However, small bile ducts significantly increase the risk of postoperative complications, such as bile leakage, stricture, and cholangitis, which can impede recovery and even necessitate re-transplantation. In an experimental study by Yang Hongqiang et al., involving porcine portoenterostomy, the safety and feasibility of portoenterostomy were demonstrated. Their pathological observations revealed excellent bile duct healing with a lower incidence of bile duct stricture compared to choledochojejunostomy (18). However, due to the larger bile duct diameters in pigs, these findings do not fully address the complication and stricture rates associated with small bile duct portoenterostomy.

In summary, most previous studies on portoenterostomy have focused on larger bile ducts, with limited discussion on the comparison between small bile duct portoenterostomy and choledochojejunostomy. Furthermore, prior studies have not included the use of immunosuppressive drugs or hormones, leaving it unclear whether these medications influence postoperative outcomes. Therefore, this study aims to compare small bile duct or multiple small bile duct-jejunal portoenterostomy with choledochojejunostomy, with the goal of determining whether portoenterostomy can be safely applied in clinical practice, particularly in LDLT patients.

## 2 Materials and methods

### 2.1 Model establishment

This study was approved by the Ethics Committee of the Central Hospital Affiliated to Shandong First Medical University (Approval No.: JNCH2021-64). During the experiment, all animals were provided humane care in accordance with the 1975 Declaration of Helsinki for Animal Scientific Research. The study was conducted in the laboratory of the Central Hospital Affiliated to Shandong First Medical University. All animals were housed in a specific pathogen-free (SPF) facility with a 12-hour light/dark cycle, controlled temperature and humidity, and had free access to food and water.

A total of 49 adult New Zealand rabbits, weighing 3 ± 1 kg, were randomly assigned to two groups: Group A (*n* = 25, control group) and Group B (*n* = 24, experimental group). Each group was further subdivided into three subgroups, with animals sacrificed at one month, two months, and three months post-surgery for specimen collection and analysis. Group A underwent choledochojejunostomy (mucosa-to-mucosa technique), while Group B underwent portoenterostomy at the hepatobiliary junction. All rabbits were fasted for 24 h before surgery and deprived of water for 12 h post-surgery. Preoperative body weight was recorded, and anesthesia was induced via a marginal ear vein injection of 1.5% pentobarbital sodium at a dose of 2 mL/kg. During surgery, vital signs were closely monitored, and prophylactic antibiotics (cefotaxime sodium, 50 mg/kg, in 100 mL of 5% glucose-sodium chloride solution) were administered intravenously at a rate of 1 mL/min.

The rabbits were secured in the supine position on the operating table, with an electrode pad and moistened gauze placed underneath to ensure proper conductivity. The surgical area, extending from approximately 2 cm above the xiphoid process to the anterior axillary line and the umbilical plane, was shaved, disinfected, and draped with sterile surgical towels. A right paramedian incision of approximately 8–10 cm was made. Skin and subcutaneous tissue were incised layer by layer using electrocautery, and gauze was used to protect the incision.

**Figure 1:**
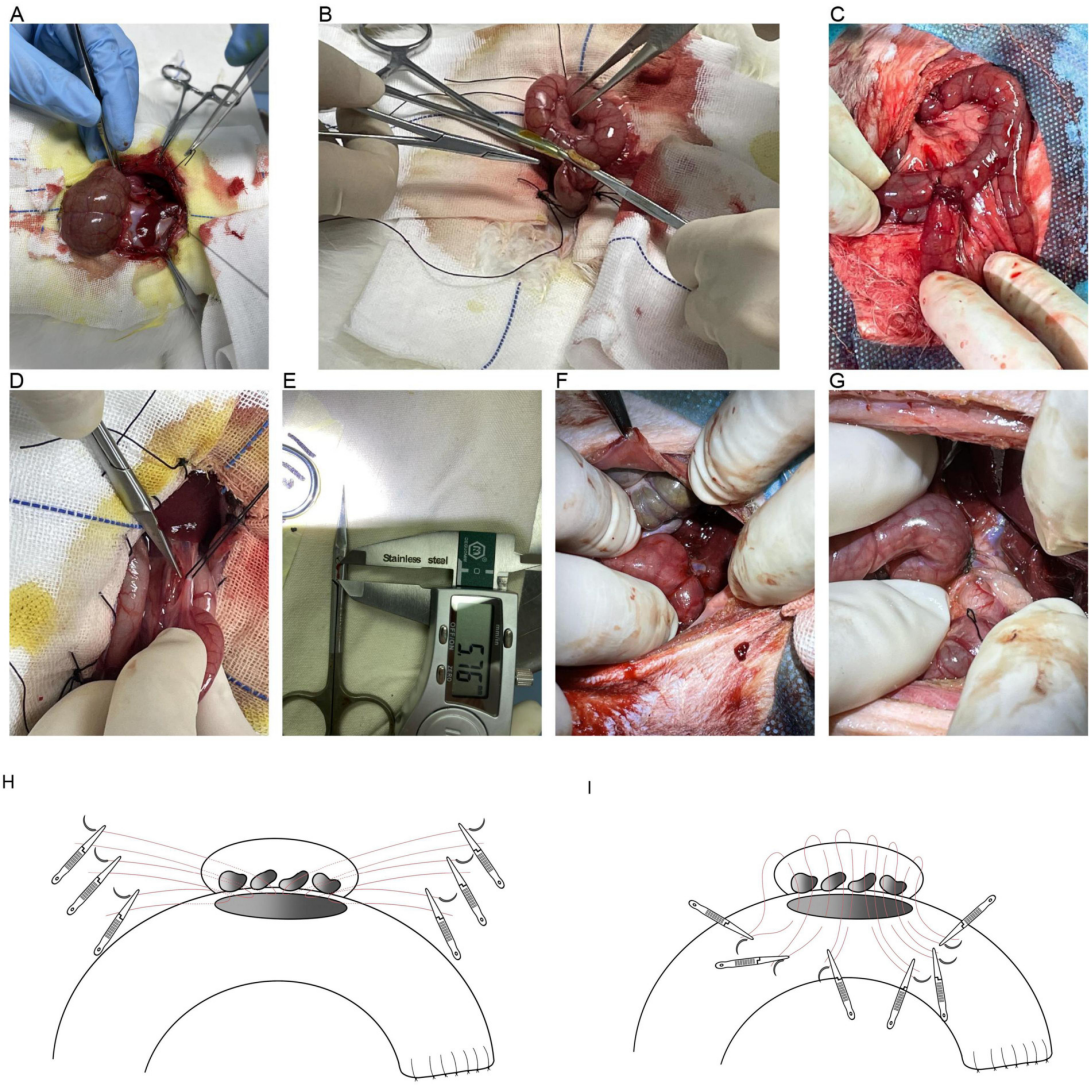
Surgical procedure for portoenterostomy **(A–I)**. **(A)** Incision of the jejunal mesentery approximately 10 cm distal to the Treitz ligament. **(B)** Suturing the distal jejunal stump. **(C)** End-to-side jejunojejunostomy performed. **(D)** Incision of the serosal layer and dissection of the common bile duct. **(E)** Measurement of bile duct wall circumference using a caliper. **(F)** Bile duct-jejunum anastomosis in Group A (Control Group) (mucosa-to-mucosa anastomosis). **(G)** Bile duct-jejunum anastomosis in Group B (Experimental Group) (bile duct fully enveloped within the jejunal incision). **(H)** Portoenterostomy (Experimental Group), anastomosis of the posterior bile duct wall and the jejunal posterior wall. **(I)** Portoenterostomy (Experimental Group), anastomosis of the jejunal anterior wall and liver tissue adjacent to the bile duct.

The stomach was elevated to expose the cecum and small intestine. The jejunal mesentery was incised approximately 10 cm distal to the ligament of Treitz, and the associated blood vessels were ligated and divided (Figure 1A). The jejunum was transected, and the distal jejunal stump was closed using continuous 5–0 silk sutures (Figure 1B), with the seromuscular layer reinforced. The distal jejunum was then mobilized and brought up, and a 1 cm transverse incision was made approximately 20 cm proximal to the transection site along the mesenteric border. An end-to-side jejunojejunostomy was performed using 6–0 Prolene sutures (Figure 1C).

### 2.2 Surgical techniques

Before bile duct anastomosis, a 3.5× magnifying loupe and headlight were used for enhanced visualization. The liver was elevated to expose the hepatoduodenal ligament. The serosal layer was incised, and the common bile duct (CBD) was isolated, ligated, and transected approximately 5 mm proximal to its entry into the duodenum (Figure 1D). The gallbladder and the proximal segment of the CBD were dissected and removed. A circumferential segment of the distal CBD was excised and opened, and the circumference of the bile duct wall was measured under tension-free conditions (Figure 1E).

For Group A, a 2 mm incision was made approximately 0.5 cm from the blind end of the distal jejunal stump, which was then anastomosed to the proximal bile duct using choledochojejunostomy (Figure 1F). Interrupted 8–0 Prolene sutures were used to close the anterior and posterior bile duct walls, ensuring the absence of bleeding and bile leakage.

For Group B, after completing the jejunojejunostomy, the distal jejunum was mobilized. A 5-mm incision was made approximately 0.5 cm from the blind end using high-frequency electrocautery, and the incision was probed and dilated with forceps to ensure a full-thickness jejunal opening. A bile duct-to-jejunal enterostomy (portoenterostomy) was performed using 8–0 Prolene single-needle sutures. Each stitch was carefully secured to ensure the bile duct was fully enveloped within the jejunal incision (Figure 1G).

Finally, the surgical site was irrigated with normal saline, and dry gauze was used to confirm the absence of bleeding and bile leakage. The abdominal cavity was closed layer by layer using 3–0 silk sutures, and the operative time was recorded.

#### 2.2.1 Establishment of fine bile duct-to-jejunal enterostomy animal model

In Group B, surgery was performed following the same procedure as Group A until completion of the jejunojejunostomy. Then, the distal jejunum was mobilized, and a 5-mm incision was made approximately 0.5 cm from the blind end using high-frequency electrocautery. The incision was dilated with forceps to ensure full-thickness opening of the jejunum (Figure 1G). Next, a bile duct-to-jejunal enterostomy (portoenterostomy) was performed using 8–0 Prolene single-needle sutures. The suture was passed through the outer side of the proximal bile duct near the cut end, then through the jejunal opening. Artery clamps were used to secure the suture. The posterior bile duct wall was sutured with 8–0 Prolene in intervals of 0.6–0.8 mm, and the suture was tied after securing with an artery clamp (Figure 1H). The anterior bile duct wall was sutured next, with the 8–0 Prolene suture passed from the outer side of the jejunum to the liver tissue above the bile duct, ensuring the bile duct was fully enveloped in the jejunal opening (Figure 1I). Each stitch was fixed with an artery clamp, and after completing the anterior wall suturing, the suture was tied sequentially. The anastomosis was completed, and the surgical site was irrigated with saline. Dry gauze was used to check for bleeding and bile leakage. Once confirmed, the abdominal cavity was closed layer by layer using 3–0 silk sutures, and the surgical time was recorded.

### 2.3 Postoperative care and sample collection

Postoperatively, immunosuppressive therapy was administered using cyclosporine A at 15 mg/kg/day mixed in drinking water, and methylprednisolone at 7 mg/kg/day ground into powder and mixed with feed. To maintain therapeutic levels of immunosuppression, cyclosporine A blood concentrations were regularly monitored, aiming for a target range of 200–400 ng/mL. The methylprednisolone dose was adjusted based on clinical responses and side effects to ensure consistent immune suppression throughout the study period. For both the control and experimental groups, rabbits in three subgroups were sacrificed under general anesthesia at one, two, and three months postoperatively, totaling 24 adult New Zealand rabbits. Rabbits were euthanized by air embolism after anesthesia. Anastomotic tissue and bile duct tissue proximal to the anastomosis were harvested and divided into three portions: one fixed in 4% formaldehyde, one fixed in glutaraldehyde, and one stored in liquid nitrogen for subsequent analysis.

### 2.4 Observation of basic indicators

Postoperatively, detailed records were maintained regarding the rabbits' dietary status, body weight changes, incidence of bile leakage, and abdominal infections. Mortality rates were also recorded, and complications were treated actively.

### 2.5 Serum and whole blood analysis

At one, two, and three months postoperatively, blood samples were collected via cardiac puncture to measure cyclosporin A (CsA) concentrations and perform other necessary serum and whole blood tests. These included measurements of serum albumin (ALB), alkaline phosphatase (ALP), alanine transaminase (ALT), aspartate transaminase (AST), total bilirubin (TBIL), direct bilirubin (DBIL), blood urea nitrogen (BUN), gamma-glutamyl transferase (GGT), total bile acid (TBA), serum creatinine (Scr), and white blood cell levels. CsA levels were specifically monitored to adjust CsA dosage as needed.

### 2.6 Statistical analysis

Statistical analysis was performed using SPSS 27.0 and GraphPad version 9.0 (GraphPad Software, La Jolla, CA, USA). For continuous variables with a normal distribution, means ± standard deviations (SD) were used, and group comparisons were conducted using independent sample t-tests. Non-normally distributed continuous data were expressed as median and interquartile range (P25, P75), and between-group comparisons were performed using the Mann–Whitney *U*-test. A *P*-value < 0.05 was considered statistically significant. All experiments were independently repeated at least three times.

## 3 Results

### 3.1 General characteristics, mortality, and morbidity statistics

A total of 49 rabbits were included in the study, with 25 in the Choledochojejunostomy group and 24 in the Portoenterostomy group. Every month, 4 rabbits from the Choledochojejunostomy group and 4 from the Portoenterostomy group were euthanized for specimen collection. During the experiment, 12 rabbits developed decreased appetite and lethargy in the mid-postoperative period and were treated with oral gentamicin.

Among the control group (choledochojejunostomy), 6 rabbits (6/25) died before the scheduled euthanasia. Similarly, in the experimental group (portoenterostomy), 4 rabbits (4/24) died before the scheduled euthanasia. In the control group, 6 rabbits died: 1 from respiratory infection, 4 from bile leakage resulting in intra-abdominal infection, and 1 from intestinal obstruction due to anastomotic stricture. In the experimental group, 4 rabbits died: 1 from wound infection, 1 from intestinal leakage, and 2 from bile leakage leading to intra-abdominal infection.

### 3.2 Serum and whole blood test results

Analysis of serum and whole blood test results revealed that on postoperative day 15, both groups exhibited significantly elevated serum total bilirubin (TBIL) and direct bilirubin (DBIL) levels compared to normal ranges. These levels decreased to near-normal ranges by postoperative day 30. However, by postoperative day 90, the mean bilirubin levels were significantly elevated in both groups, likely due to the development of anastomotic stenosis and cholangitis in some rabbits. The average alkaline phosphatase (ALP) levels followed a similar trend in both groups. Additionally, mean serum creatinine (Scr) levels were near-normal on postoperative day 15 but significantly increased by postoperative day 90, suggesting potential renal impairment in some rabbits.

**Figure 2:**
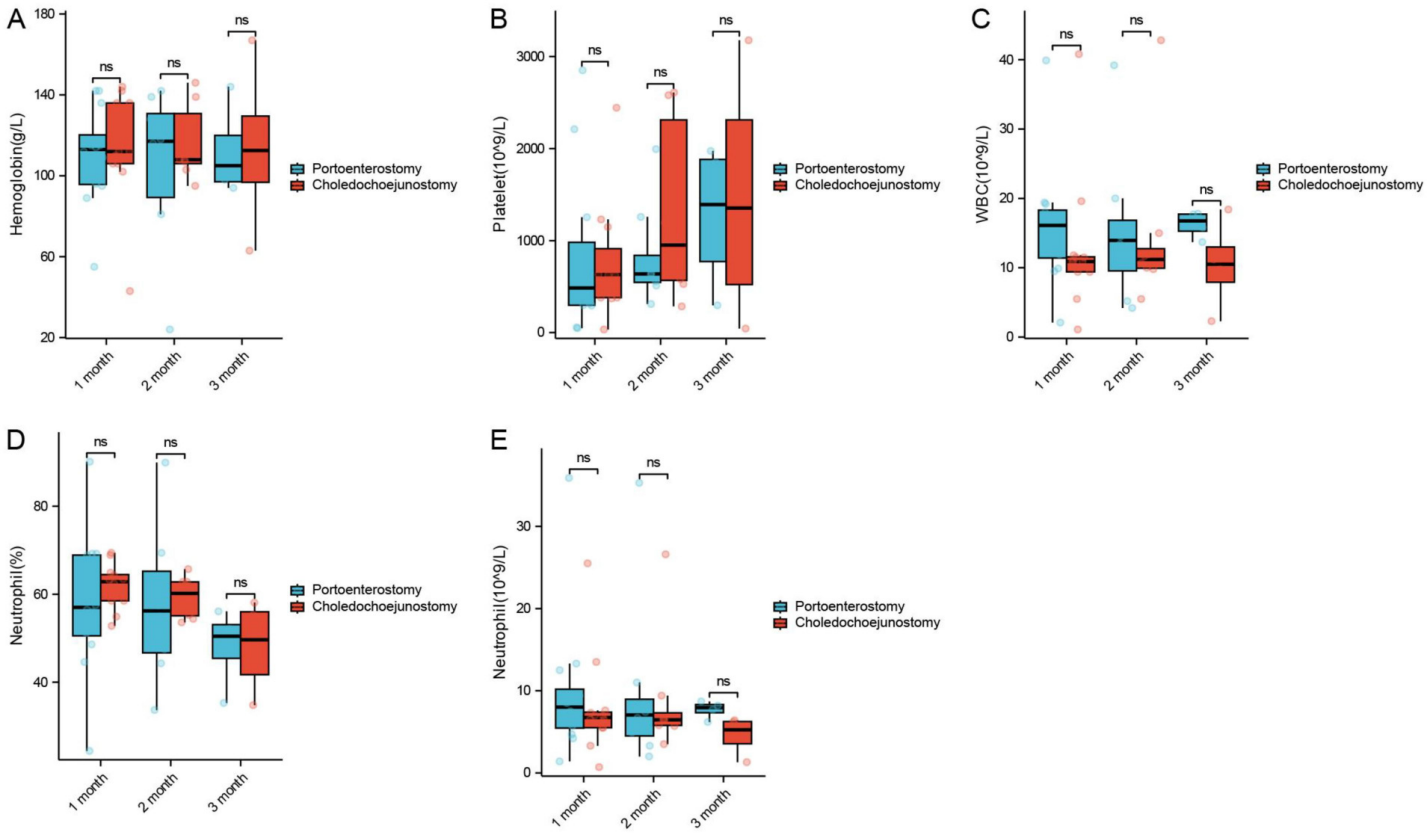
Comparison of changes of hemoglobin **(A)**, platelet **(B)**, WBC **(C)**, and neutrophil **(D,E)** in experimental rabbits at different postoperative periods.

**Figure 3:**
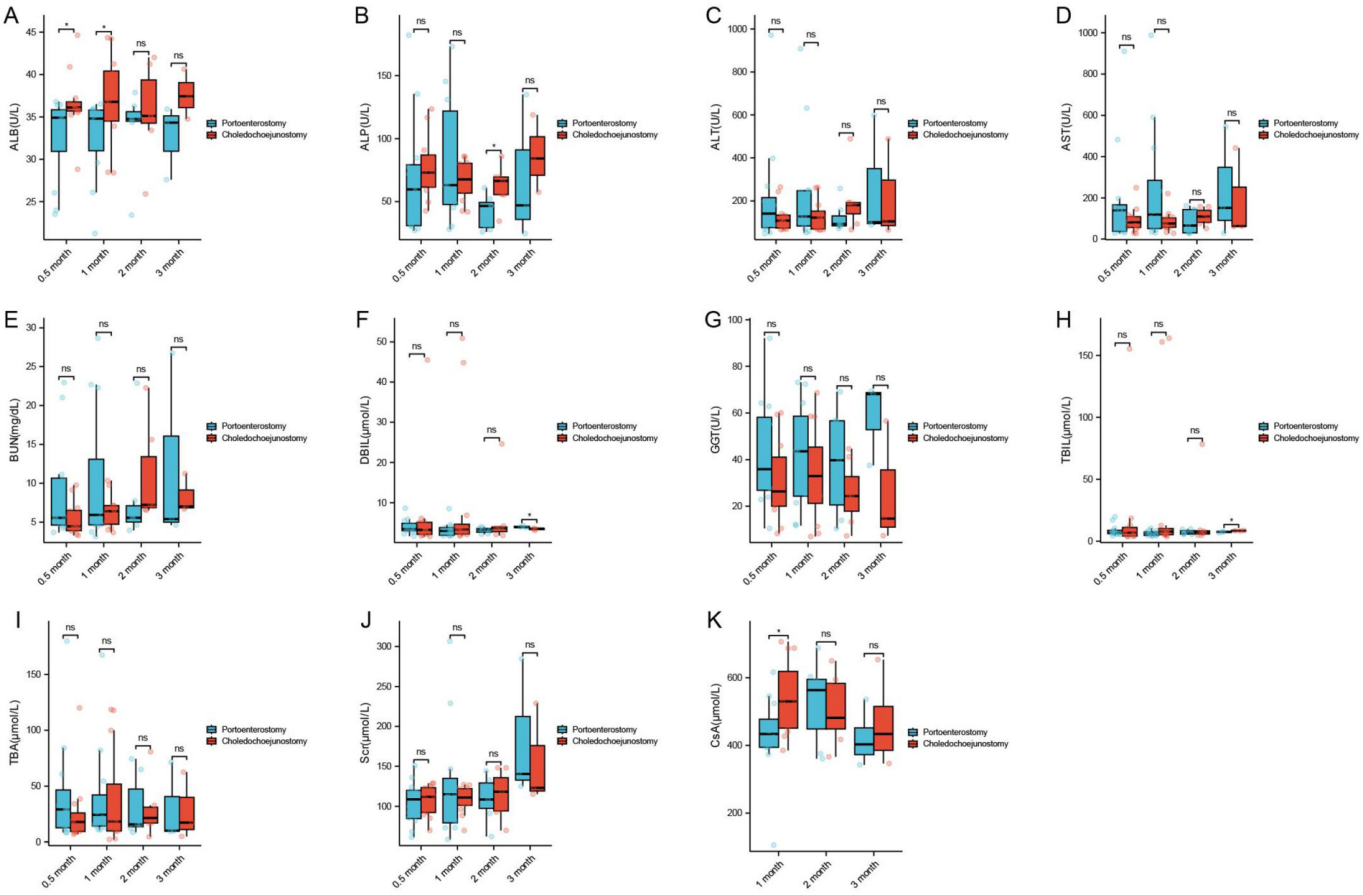
Comparison of changes of ALB **(A)**, ALP **(B)**, ALT **(C)**, AST **(D)**, BUN **(E)**, DBIL **(F)**, GGT **(G)**, TBIL **(H)**, TBA **(I)**, Scr **(J)**, and CsA **(K)** in experimental rabbits at different postoperative periods.

The experimental group showed a significant increase in WBC at 1 and 3 months postoperatively (*P* < 0.05), with no significant differences observed in other complete blood count parameters between the experimental and control groups (Figure 2, Table 1). Additionally, the experimental group (portoenterostomy) had lower levels of ALB at 1 and 3 months, ALP at 2 months, and CsA at 1 month compared to the control group (choledochojejunostomy) (*P* < 0.05) (Figure 3, Table 1).

**Table 1:** Comparison of changes in hemoglobin, platelet, WBC, neutrophil, ALB, ALP, ALT, AST, BUN, DBIL, GGT, TBIL, TBA, Scr, and CsA at different postoperative periods.

Variable	time	t/Z	*P*-value	Variable	time	t/Z	*P*-value
Hemoglobin	1 month	−1.410	.172	AST	1 month	−1.328	.184
	2 month	−1.390	.178		2 month	−.115	.908
	3 month	−1.146	.264		3 month	−1.039	.299
Platelet	1 month	−.520	.603	BUN	1 month	−.173	.862
	2 month	-.462	.644		2 month	−.115	.908
	3 month	-.462	.644		3 month	−.520	.603
WBC	1 month	−2.022	**.** **043**	DBIL	1 month	−.924	.356
	2 month	−1.617	.106		2 month	−.577	.564
	3 month	−2.310	.**021**		3 month	−.493	.622
Neutrophil (%)	1 month	−.718	.485	GGT	1 month	1.070	.296
	2 month	−1.062	.308		2 month	1.370	.196
	3 month	−1.854	.077		3 month	1.733	.158
Neutrophil	1 month	.604	.552	TBIL	1 month	−1.532	.154
	2 month	.306	.764		2 month	-.865	.397
	3 month	2.424	.052		3 month	−1.062	.300
ALB	1 month	−2.096	.**048**	TBA	1 month	.117	.908
	2 month	−1.573	.130		2 month	1.571	.136
	3 month	−2.886	.**009**		3 month	1.552	.135
ALP	1 month	.964	.352	Scr	1 month	1.043	.317
	2 month	−2.642	.**021**		2 month	.315	.756
	3 month	−.472	.662		3 month	.646	.525
ALT	1 month	−.635	.525	CsA	1 month	−2.444	.**023**
	2 month	−.289	.773		2 month	.448	.661
	3 month	−.462	.644		3 month	−.579	.584

### 3.3 Comparison of anastomotic stenosis

**Figure 4:**
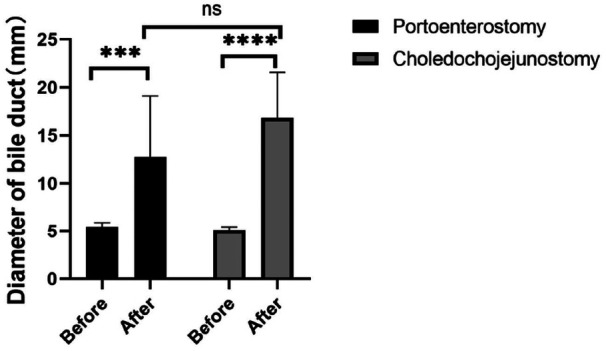
Differences between diameter of bile duct between treatment and control groups. Bar chart of the difference in diameter of bile duct between treatment and control groups, measured at the time of euthanasia for each of the three subgroups (1, 2, and 3 months postoperatively).

**Figure 5:**
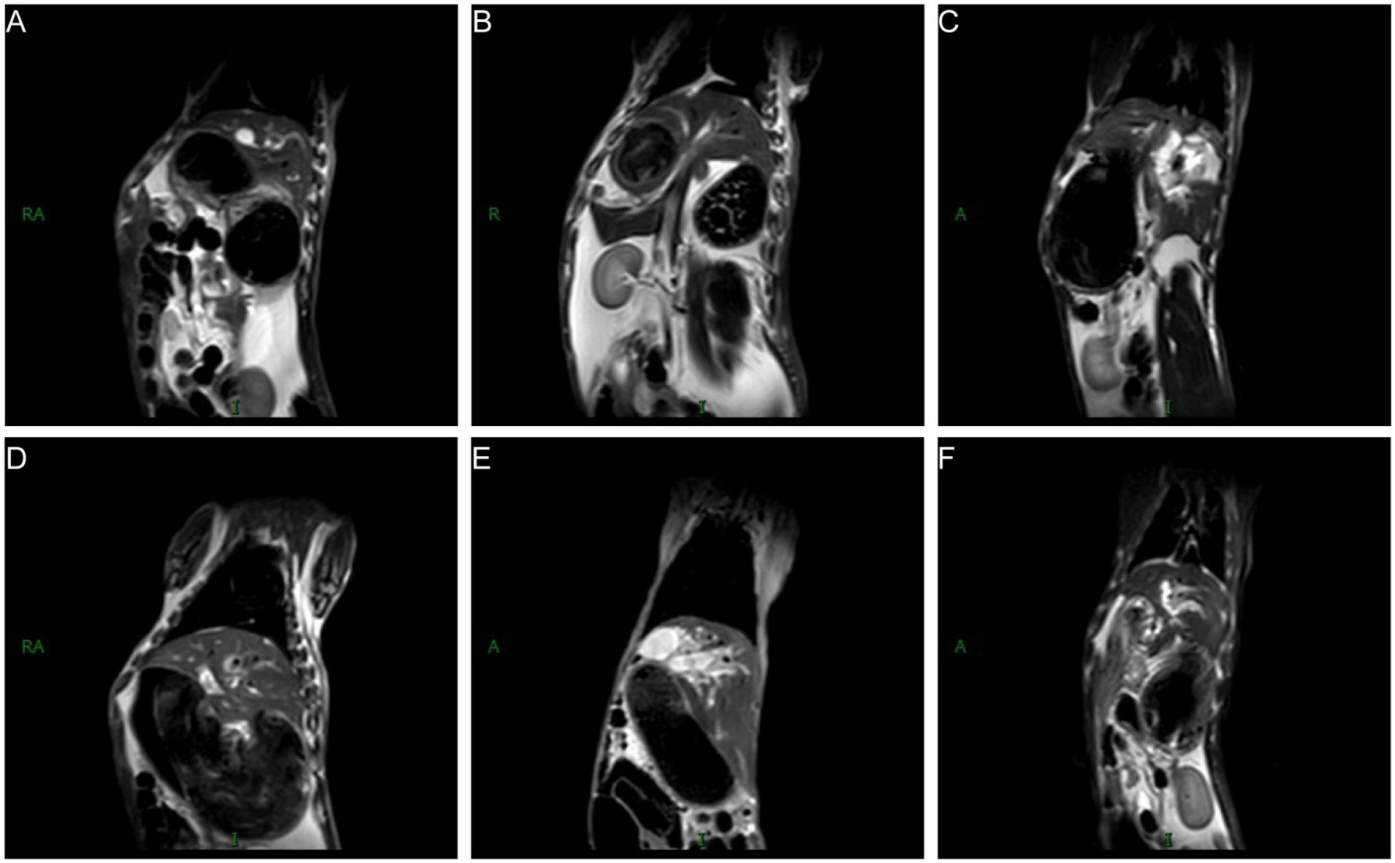
Differences in MRCP between treat and control groups. MRCP in 1 **(A)**, 2 **(B)** and 3 **(C)** months after choledochojejunostomy. MRCP in 1 **(D)**, 2 **(E)** and 3 **(F)** months after portoenterostomy.

The postoperative bile duct diameter in both groups was significantly larger than the preoperative diameter; however, no significant differences were observed in bile duct diameters between the experimental and control groups at various postoperative stages (*P* > 0.05) (Figure 4). Different degrees of anastomotic stenosis were observed at various postoperative time points. Magnetic resonance cholangiopancreatography (MRCP) further revealed significant intrahepatic bile duct dilation in both groups postoperatively (Figure 5).

### 3.4 Histological analysis with HE and masson staining

#### 3.4.1 Control group (choledochojejunostomy)

**Figure 6:**
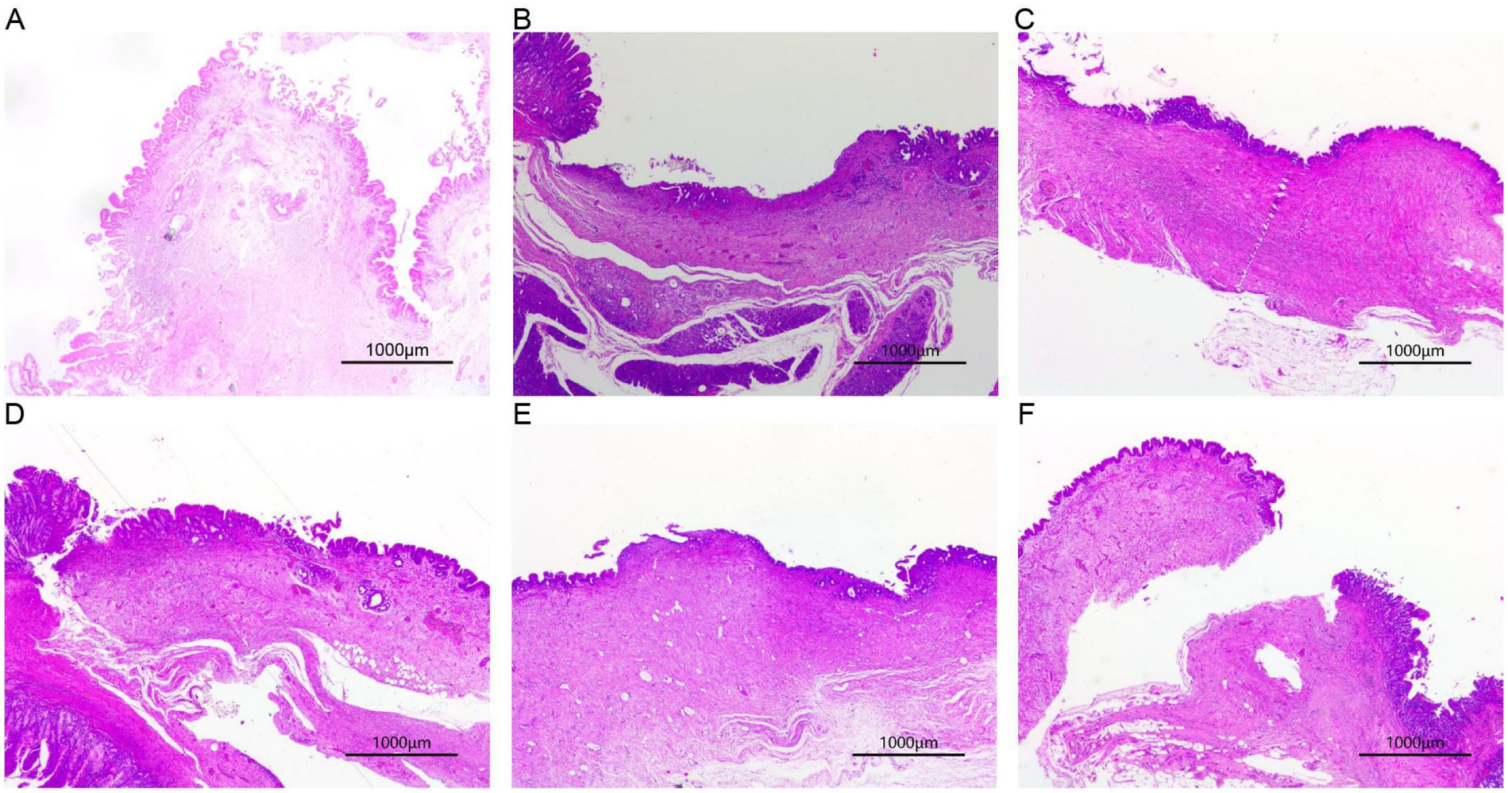
HE staining of anastomotic tissues from treat and control groups. HE staining of anastomotic tissue 1 **(A)**, 2 **(B)** and 3 **(C)** months after choledochojejunostomy. HE staining of anastomotic tissue 1 **(D)**, 2 **(E)** and 3 **(F)** months after portoenterostomy.

HE staining at one month postoperatively revealed thickening of the bile duct and intestinal wall at the anastomotic site, along with proliferation of fibrous connective tissue, hyaline and mucinous degeneration, and scar formation. Epithelial proliferation was observed in both the bile duct and intestine, accompanied by minimal lymphocytic and plasma cell infiltration. Mononuclear and multinucleated giant cells aggregated at the suture sites, with focal lymphocyte infiltration in the surrounding intestinal mucosa and localized neutrophil infiltration. By two months postoperatively, the anastomotic site had healed with fibrous scar formation. Masson staining showed green-stained fibrous tissue and red-stained muscular mucosa. Epithelial growth from the bile duct and intestine was continuous, with pronounced intestinal mucosal proliferation and minimal lymphocyte infiltration. By three months, the bile duct and intestine were connected by fibrous scars with good healing. Masson staining revealed no red-stained muscular mucosa at the scar surface, where bile duct and intestinal epithelium grew together (Figure 6).

#### 3.4.2 Experimental group (portoenterostomy)

**Figure 7:**
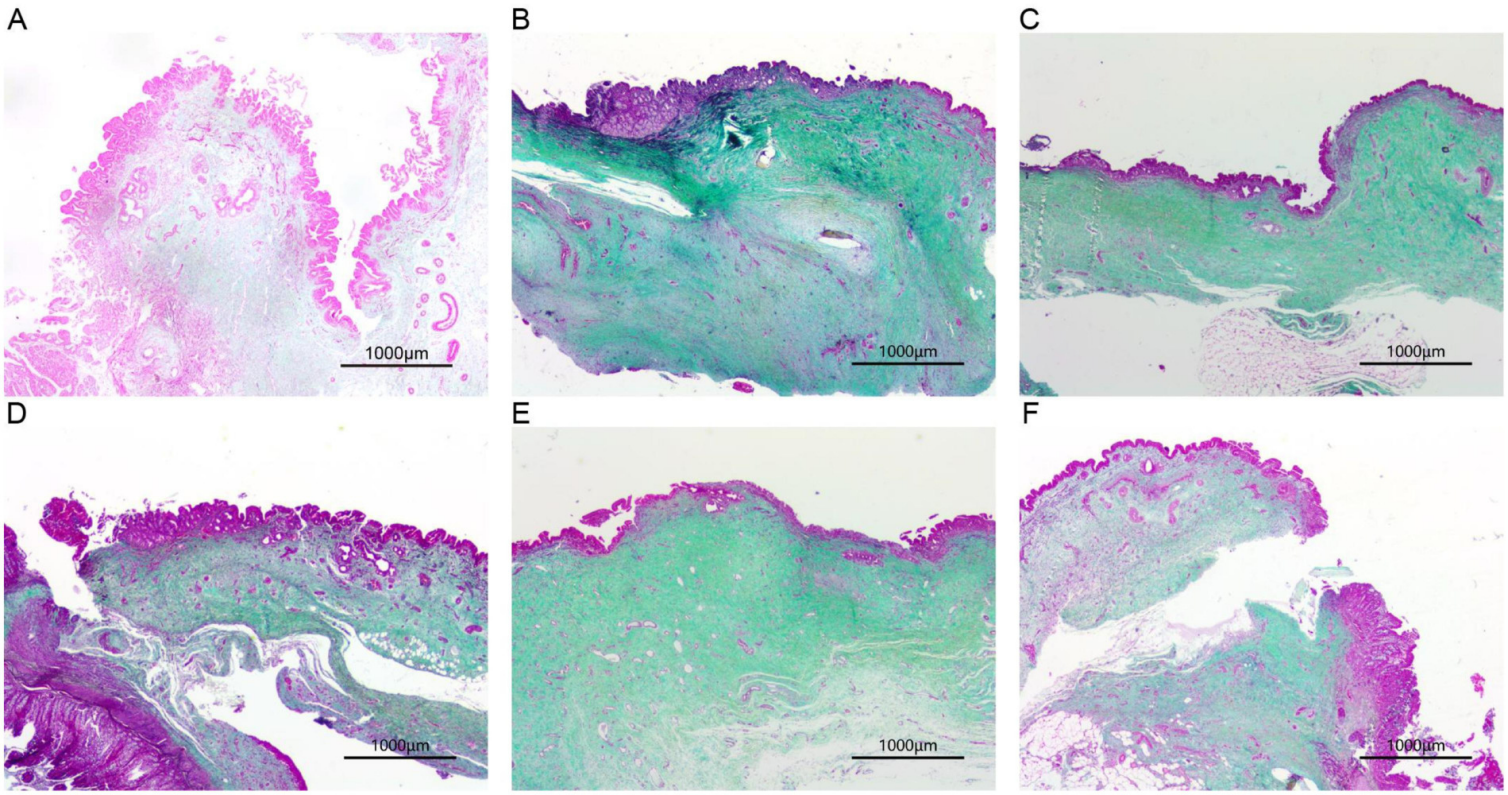
Masson staining of anastomotic tissues from treat and control groups. Masson staining of anastomotic tissue 1 **(A)**, 2 **(B)** and 3 **(C)** months after choledochojejunostomy. Masson staining of anastomotic tissue 1 **(D)**, 2 **(E)** and 3 **(F)** months after portoenterostomy.

At one month postoperatively, the anastomotic site showed good healing with mild erosion and scattered lymphocyte and neutrophil infiltration in the bile duct and intestinal mucosa. Epithelial growth was continuous between the bile duct and jejunum. The surrounding bile duct and intestinal mucosa exhibited significant lymphocyte and neutrophil infiltration, localized lymphocyte infiltration on the serosal surface, and granulation tissue proliferation with inflammatory exudates. By two months, the anastomotic site was connected by fibrous scars with good healing. Masson staining showed discontinuous red-stained muscular mucosa, and epithelial growth from both the bile duct and jejunum was evident at the scar surface. By three months, the anastomotic site was connected by fibrous scars with continuous epithelial growth from the bile duct and jejunum, although no distinct anastomotic site was observed (Figure 7).

Histological analysis with HE and Masson staining revealed no significant pathological differences in anastomotic healing between the two surgical techniques.

## 4 Discussion

The lack of awareness regarding organ donation and the shortage of liver grafts have resulted in many end-stage liver disease patients either dying or missing transplantation opportunities, thus driving the development of living donor liver transplantation (LDLT). While laparoscopic donor hepatectomy has advanced significantly, challenges remain in performing cholangiography, leading to higher bile duct transection planes. Consequently, multiple small bile ducts may be present on the hepatic cut surface, increasing the difficulty of surgical reconstruction due to limited operating space. The technical difficulty is further exacerbated as the bile duct diameter decreases (33, 34). Portoenterostomy, originally developed for treating biliary atresia in children (19), has since been adapted for radical surgery of hilar cholangiocarcinoma. This study compares choledochojejunostomy (control group) and portoenterostomy (experimental group) in New Zealand rabbits to evaluate the safety and efficacy of these techniques in human LDLT, particularly under the administration of immunosuppressants and steroids.

This study evaluated the safety and feasibility of portoenterostomy and choledochojejunostomy in New Zealand rabbits undergoing biliary reconstruction under immunosuppressive conditions. Our results, based on 73 rabbits, demonstrated that portoenterostomy is technically safe and reliable, with comparable postoperative mortality and complication rates to choledochojejunostomy. These findings suggest that portoenterostomy could be a safe alternative for biliary reconstruction following liver surgery, particularly with the adjunct use of immunosuppressive therapy. Key surgical details, such as precise suturing techniques, accurate anastomotic alignment, and meticulous postoperative monitoring, were identified as critical factors for successful outcomes.

The high postoperative mortality and complication rates observed in both groups, particularly bile leakage, may be attributable to the following factors: (1) The thin and fragile bile duct wall in rabbits is prone to tearing during suturing, and bile duct leakage is exacerbated by the operator's technical expertise, ultimately leading to intra-abdominal infection and death. (2) Both groups received immunosuppressants (cyclosporine A) and steroids postoperatively, potentially inducing immunosuppression that predisposes rabbits to respiratory and abdominal infections. Although the symptoms of some animals improved with cessation of cyclosporine A and anti-inflammatory treatments, others succumbed to these complications, resulting in high postoperative mortality.

Interestingly, the degree of anastomotic stenosis was lower in the portoenterostomy group compared to the choledochojejunostomy group. This could be attributed to the bile duct epithelium migrating toward the jejunal epithelium, causing eversion of the bile duct opening in the portoenterostomy technique (18). Following epithelialization of the wound surface, granulation tissue proliferation is suppressed, and scar formation ceases (35–38). Additionally, the larger jejunal opening and wound area between the jejunum and bile duct in the portoenterostomy technique reduce the likelihood of stenosis compared to the smaller diameter of choledochojejunostomy, which is further constrained by the thickness of the jejunal wall and excessive tissue protrusion at the anastomotic site. The predominantly elastic fiber composition of the extrahepatic bile duct, with a fibrous tissue-rich submucosa and low smooth muscle content, predisposes bile duct transection sites to fibrotic contraction and scar formation due to excessive fibroblast activity and collagen synthesis (39). Despite these challenges, the use of immunosuppressants did not negatively impact the healing process, providing valuable insights for clinical applications.

Elevated serum bilirubin and GGT levels postoperatively suggest the occurrence of anastomotic stenosis and cholangitis in some rabbits. Cholangitis, a common complication following bile duct injury or anastomosis, is primarily caused by poor bile drainage due to stenosis and retrograde bacterial migration from the intestine. Anastomotic alterations disrupt the natural physiological pathway of bile, compromising the anti-reflux function of the sphincter of Oddi and promoting bacterial translocation into the biliary system. This bacterial migration exacerbates local inflammation and infection, further impairing anastomotic healing (29, 40, 41). Additionally, changes in serum creatinine levels indicate potential renal dysfunction in some rabbits, possibly related to postoperative drug administration (42). These findings emphasize the importance of monitoring and preventing these potential complications in postoperative management.

Histopathological examination revealed no significant differences in healing outcomes between portoenterostomy and choledochojejunostomy during the 1–3 month postoperative period. Both techniques demonstrated favorable healing processes, although varying degrees of inflammation and scar formation were observed. These results underscore the importance of anti-inflammatory treatment and scar control in postoperative management.

Finally, the findings of this study hold significant implications for LDLT. Biliary anatomical variations are common in living donors, and small bile duct diameters further complicate traditional anastomotic techniques, increasing complication rates. Portoenterostomy offers a promising alternative that may enhance postoperative recovery and reduce the incidence of complications. Additionally, this technique has the potential to shorten hospital stays and reduce healthcare costs.

Despite providing valuable preliminary data, this study has several limitations. First, the relatively small sample size may limit the generalizability of the results. Second, the study was conducted in an animal model, and the high mortality rates observed in rabbits may not accurately reflect outcomes in human bile duct reconstruction. The results require further validation in clinical trials. Additionally, the elevated bilirubin levels seen in some rabbits could suggest potential issues with the surgical technique, especially in the handling of the common bile duct and anastomosis. Complications such as bile duct injury or bile leakage are relatively common in small animal models and may have contributed to the increased bilirubin levels observed postoperatively. Moreover, the lack of long-term follow-up data limits the evaluation of the long-term efficacy of these techniques. Future studies should involve larger clinical trials with extended follow-up periods to assess the long-term safety and outcomes of portoenterostomy for small bile ducts. Additionally, exploring the mechanisms of immunosuppressive drugs and steroids in bile duct anastomosis, as well as optimizing postoperative management, will be crucial research directions moving forward.

## 5 Conclusion

This study provides scientific evidence supporting the clinical application of portoenterostomy, demonstrating its potential in biliary reconstruction during hepatobiliary surgery. With further research and clinical validation, this surgical technique holds promise as a vital approach for biliary reconstruction. Additionally, the use of immunosuppressive agents does not have a significant impact on anastomotic healing, although further investigation is needed.

## Data Availability

The original contributions presented in the study are included in the article/Supplementary Material, further inquiries can be directed to the corresponding author.
